# Supplementary material for: Exploring the basis of 2-propenyl and 3-butenyl glucosinolate synthesis by QTL mapping and RNA-sequencing in Brassica juncea
Source: PLoS One. 2019 Oct 18;14(10):e0220597. doi: 10.1371/journal.pone.0220597 (PMC6799926; doi:10.1371/journal.pone.0220597)
Supplement: S1 Table — (DOCX) [file pone.0220597.s001.docx]

**S1 Table: The synteny analysis between unique transcript of ILP primers and the Brapa_1.0 genome.**

| **Serial Number** | **ILP Markers on LG08 developed from *B. napus* and *B. rapa*** | **Primers** | **Unique Transcript-fragment ID** | **Mapped chromosomes in *Brassica rapa* cultivar Chiifu-401-42 (Brapa_1.0) genome** | [**E value**](https://blast.ncbi.nlm.nih.gov/Blast.cgi?CMD=Get&ALIGNMENTS=100&ALIGNMENT_VIEW=Pairwise&BLAST_SPEC=OGP__3711__12578&DATABASE_SORT=0&DESCRIPTIONS=100&DYNAMIC_FORMAT=on&FIRST_QUERY_NUM=0&FORMAT_OBJECT=Alignment&FORMAT_PAGE_TARGET=&FORMAT_TYPE=HTML&GET_SEQUENCE=yes&I_THRESH=&LINE_LENGTH=60&MASK_CHAR=2&MASK_COLOR=1&NUM_OVERVIEW=100&PAGE=MegaBlast&QUERY_INDEX=0&QUERY_NUMBER=0&RESULTS_PAGE_TARGET=&RID=BU5PC7EA015&SHOW_LINKOUT=yes&SHOW_OVERVIEW=yes&STEP_NUMBER=&OLD_VIEW=false&DISPLAY_SORT=0&HSP_SORT=0) |
| --- | --- | --- | --- | --- | --- |
| 1 | BnapPIP86 | F: 5’-AAGAAGGCGAAGACTGACGA-3’  R: 5’-TATCGCCACGCTTGTCATAG-3’ | PUT-155a-Brassica_napus-22815 | A8 | 2.00E-80 |
| 2 | BrapPIP38 | F: 5’-AACCCCCTCGTCTCAAGTCT-3’  R: 5’-TACCAGGCTTCCTGGGTACA-3’ | PUT-155a-Brassica_rapa-4412 | A8 | 6.00E-125 |
| 3 | BrapPIPR3 | F: 5’-AAAAGGCAGGGGAGATGATT-3’  R: 5’-CAGATCCCAACAGCGTTCAT-3’ | PUT-155a-Brassica_rapa-1373 | A1 | 5.00E-131 |
| 4 | BnapPIP182 | F: 5’-ACCGGTGTCAAGAGGCTTTA-3’  R: 5’-CCACAGATAACCGCAACCTT-3’ | PUT-155a-Brassica_rapa-1609 | A8 | 3.00E-121 |
| 5 | BnapPIP1628 | F: 5’-GCGAAATCCGCTCTAGAATC-3’  R: 5’-CTTCAGCAGTGACAAAATCAGG-3’ | PUT-155a-Brassica_napus-8901 | A8 | 3.00E-163 |
| 6 | BnapPIP1621 | F: 5’-GCCTGTTCGTCTACCACCAC-3’  R: 5’-ACTGCGACGATTCTTCATCC-3’ | PUT-155a-Brassica_napus-7002 | A8 | 4.00E-131 |
| 7 | BrapPIPR31 | F: 5’-AAATGTGAGCCTCAGGGACT-3’  R: 5’-TGCAGGCTTCTTGTTGTGAT-3’ | PUT-155a-Brassica_rapa-5039 | A7 | 5.00E-84 |
| 8 | BrapPIP89 | F: 5’-AAGCAAGGGAAGATCAACGA-3’  R: 5’-AACATCTTGGAAGGGACCTG-3’ | PUT-155a-Brassica_rapa-9044 | A8 | 5.00E-61 |
| 9 | BnapPIP1371 | F: 5’-GAAGTTGCAGGATGGTTGGT-3’  R: 5’-CGACATCTTGGAATGGACCT-3’ | PUT-155a-Brassica_napus-2821 | A8 | 2.00E-100 |
| 10 | BnapPIP1300-1 | F: 5’-CTTGGCTGAGTTCAAGATCG-3’  R: 5’-CGGAGAATTGAGGATCTCGT-3’ | PUT-155a-Brassica_napus-5410 | A8 | 0 |
| 11 | BnapPIP1848-3 | F: 5’-GGCGAGAGTGATCCTTCAGA-3’  R: 5’-TGACTGGCTTCTCACCAAAG-3’ | PUT-155a-Brassica_napus-10135 | A8 | 2.00E-55 |
| 12 | BnapPIP1573 | F: 5’-GCATAACTCGTTGGGGACAG-3’  R: 5’-CGGTGGCTAGCATTGACTTC-3’ | PUT-155a-Brassica_napus-10887 | A8 | 6.00E-61 |
| 13 | BnapPIP1854 | F: 5’-GGCTATCAAACGAGCAGGAG-3’  R: 5’-CACCACTGATGCATGGTTCT-3’ | PUT-155a-Brassica_napus-4048 | A5 | 1.00E-174 |
| 14 | BnapPIP137 | F: 5’-GGCTATCAAACGAGCAGGAG-3’  R: 5’-CACCACTGATGCATGGTTCT-3’ | PUT-155a-Brassica_napus-4048 | A5 | 1.00E-174 |
| 15 | BnapPIP1848-2 | F: 5’-GGCGAGAGTGATCCTTCAGA-3’  R: 5’-TGACTGGCTTCTCACCAAAG-3’ | PUT-155a-Brassica_napus-10135 | A8 | 2.00E-55 |
| 16 | BnapPIP870 | F: 5’-CCCTAAGCCTCCTAGGTCGT-3’  R: 5’-TCCTCCCTCATGAACTGCTT-3’ | PUT-155a-Brassica_napus-7356 | A8 | 2.00E-80 |
| 17 | BnapPIP668-2 | F: 5’-CACTACTTGGAGCCCACTCG-3’  R: 5’-GAGCCAAGGTCACACCTAGC-3’ | PUT-155a-Brassica_napus-7192 | A8 | 2.00E-121 |
| 18 | BnapPIP1462 | F: 5’-GAGGCTGAGCGTCTTTTCAC-3’  R: 5’-CGAGGCACTTGAAGACTGCT-3’ | PUT-155a-Brassica_napus-239 | A8 | 1.00E-108 |
| 19 | BnapPIP1848-1 | F: 5’-GGCGAGAGTGATCCTTCAGA-3’  R: 5’-TGACTGGCTTCTCACCAAAG-3’ | PUT-155a-Brassica_napus-10135 | A8 | 2.00E-55 |
| 20 | BnapPIP88 | F: 5’-AAGAATCGGTCGTTTGGTTG-3’  R: 5’-CAGTGACTGGCTTCTCACCA-3’ | PUT-155a-Brassica_napus-7534 | A8 | 7.00E-98 |
| 21 | BnapPIP592 | F: 5’-CAACGTCGAGCACCATGTC-3’  R: 5’-ACCAATCATAGCCCTGCAAC-3’ | PUT-155a-Brassica_napus-7209 | A8 | 0 |
| 22 | BrapPIP1580 | F: 5’-GGTGAAGCTGGAGCTGACTT-3’  R: 5’-CGTGCTCGTTAACACCAACA-3’ | PUT-155a-Brassica_rapa-4357 | A8 | 2.00E-46 |
