# Supplementary material for: Exploring the basis of 2-propenyl and 3-butenyl glucosinolate synthesis by QTL mapping and RNA-sequencing in Brassica juncea
Source: PLoS One. 2019 Oct 18;14(10):e0220597. doi: 10.1371/journal.pone.0220597 (PMC6799926; doi:10.1371/journal.pone.0220597)
Supplement: S2 Table — (DOCX) [file pone.0220597.s002.docx]

**S2 Table**. The candidate genes involved in biosynthesis of and transporting of aliphatic GSLs.

| **SN^a^** | **Gene Name** | **AGI code** | **Unigene ID from RNA-Seq** | **Function** |
| --- | --- | --- | --- | --- |
| 1 | CYP83A1 | AT4G13770 | LOC106447562(U)^b^;LOC106391682(U) | aldoxime→s-alkyl-thiohydroximate [54-56] |
| 2 | SOT18 | AT1G74090 | LOC106366617(D);LOC106354324(D);LOC106436726(D) | PAPS-dependent sulfation of desulfo-GSLs→GSLs [57] |
| 3 | SOT17 | AT1G18590 |  |  |
| 4 | IIL1 | AT4G13430 | LOC106416451(U) | 2-Alkyl-malic acid→3-Alkyl-malic acid [55,58] |
| 5 | IPMI2 | AT2G43100 | LOC106434491(U) |  |
| 6 | IPMI SSU1 | AT2G43090 |  |  |
| 7 | AOP3 | AT4G03050 | LOC106430050(U);LOC106438719(U);LOC106389979(U) | methylsulfinylalkyl GSL→ hydroxyalkyl GSL [55] |
| 8 | SUR1 | AT2G20610 | LOC106440999(D) | s-alkyl-thiohydroximate→ thiohydroximate [55,59,60] |
| 9 | MYB28 | AT5G61420 | LOC106382207(U);LOC106429668(U) | the whole process of biosynthesis of methionine-derived GSL [54] |
| 10 | MYB29 | AT5G07690 |  |  |
| 11 | MYB76 | AT5G07700 |  |  |
| 12 | GTR2 | AT5G62680 | LOC106411192(U);LOC106347844(D) | GSL transporting [61] |
| 13 | GTR1 | AT3G47960 |  |  |

^a^: Serial Number.

^b^: “U” means up-regulated, “D” means down-regulated.
