# Supplementary material for: Exploring the basis of 2-propenyl and 3-butenyl glucosinolate synthesis by QTL mapping and RNA-sequencing in Brassica juncea
Source: PLoS One. 2019 Oct 18;14(10):e0220597. doi: 10.1371/journal.pone.0220597 (PMC6799926; doi:10.1371/journal.pone.0220597)
Supplement: S4 Table — (DOCX) [file pone.0220597.s004.docx]

| **SN^a^** | **Gene Name** | **AGI code** | **Unigene ID from RNA-Seq** | **Function** |
| --- | --- | --- | --- | --- |
| 1 | CYP81F1 | AT4G37430 | LOC106440059(D)^b^ | Convert indolyl-3-methyl GSL into 4-methoxyindol-3-ylmethyl GSL [[1](#_ENREF_1)] |
| 2 | CYP81F3 | AT4G37400 |  | Convert indolyl-3-methyl GSL into 4-methoxyindol-3-ylmethyl GSL[[2](#_ENREF_2)] |
| 3 | CYP81F4 | AT4G37410 |  | Convert indolyl-3-methyl GSL into 1-hydroxyindol-3-ylmethyl GSL[[2](#_ENREF_2)] |
| 4 | CYP81D7 | AT2G23190 | LOC106421607(D)；LOC106371130(D) | Indole glucosinolate metabolic process (https://www.arabidopsis.org/index.jsp) |
| 5 | CYP81D6 | AT2G23220 |  |  |
| 6 | CYP81D5 | AT4G37320 |  |  |
| 7 | CYP81D4 | AT4G37330 |  |  |
| 8 | CYP82C4 | AT4G31940 | LOC106390013(U) | Indole glucosinolate metabolic process  (https://www.arabidopsis.org/index.jsp) |
| 9 | CYP82C2 | AT4G31970 |  |  |
| 10 | CYP82C3 | AT4G31950 |  |  |
| 11 | ESP | AT1G54040 | LOC106415866(U);LOC106345821(U) | Breakdown of GLS[[2](#_ENREF_2)] |
| 12 | ESM1 | AT3G14210 | LOC106439503(D) | Represses nitrile formation and favors isothiocyanate production during glucosinolate hydrolysis[[3](#_ENREF_3)] |
| 13 | MBP2 | AT1G52030 | LOC106428582(D) | Involved in the activation of myrosinase for breackdown of GLS[[4](#_ENREF_4)] |

**S4 Table: The candidate genes involved in biosynthesis of indolic GLS.**

^a^: Serial Number.

^b^: “U” means up-regulated, “D” means down-regulated.

**Reference:**

1. Chiu Y-C, Juvik JA, Ku K-M (2018) Targeted Metabolomic and Transcriptomic Analyses of “Red Russian” Kale (Brassicae napus var. pabularia) Following Methyl Jasmonate Treatment and Larval Infestation by the Cabbage Looper (Trichoplusia ni Hübner). International Journal of Molecular Sciences 19: 1058.

2. Liu S, Liu Y, Yang X, Tong C, Edwards D, et al. (2014) The Brassica oleracea genome reveals the asymmetrical evolution of polyploid genomes. Nature Communications 5: 3930.

3. Burow M, Zhang ZY, Ober JA, Lambrix VM, Wittstock U, et al. (2008) ESP and ESM1 mediate indol-3-acetonitrile production from indol-3-ylmethyl glucosinolate in Arabidopsis. Phytochemistry 69: 663-671.

4. Lee JG, Lim S, Kim J, Lee EJ (2017) The mechanism of deterioration of the glucosinolate-myrosynase system in radish roots during cold storage after harvest. Food Chem 233: 60-68.
