# Supplementary material for: Exploring the basis of 2-propenyl and 3-butenyl glucosinolate synthesis by QTL mapping and RNA-sequencing in Brassica juncea
Source: PLoS One. 2019 Oct 18;14(10):e0220597. doi: 10.1371/journal.pone.0220597 (PMC6799926; doi:10.1371/journal.pone.0220597)
Supplement: S5 Table — (DOCX) [file pone.0220597.s005.docx]

**S5 Table: The primers for qRT-PCR validation.**

| **Serial Number** | **Genes** | **Forward primer (5'->3')** | **Reverse primer (5'->3')** |
| --- | --- | --- | --- |
| 1 | LOC106347844(D)^a^ | CCAACTTGTCTTCGGGAGCC | CTGCGTGATTTTCTTTGCTGGA |
| 2 | LOC106354324(D) | CGTACCGAACCAAGACGAGG | ACCAGTGACCACCGTACTCTA |
| 3 | LOC106366617(D) | AAACGTGGCGATCAGGTCTT | GATGAGTTGATGACAACAATGACA |
| 4 | LOC106382207(U) | TTCTCCGACACAACATGGCT | GGGTAAGACCCAAGAGCGTTT |
| 5 | LOC106389979(U) | GAAGCACCCACGTCTCTTCA | GAACGAGCTCTACGACCAGC |
| 6 | LOC106391682(U) | CAAATCGTCAAGAACGTAGCCA | CGGTATGGCCGCTGATAACT |
| 7 | LOC106411192(U) | TACGATGGCTCTGTTACGGC | GGCATGACTTTCCAGCCTCT |
| 8 | LOC106416451(U) | GATAAGCGTGCAAACCGCAA | TCTTCTGGCCTTCAATGACCT |
| 9 | LOC106429668(U) | ACAAGCCACTTGCCTCTACC | ACTCTGGAGACATGGAGCCT |
| 10 | LOC106430050(U) | AGAAAGACGCACATCACTGCT | ACACTGAGGGTGGTCGTAGA |
| 11 | LOC106434491(U) | ATGCGAGGAAGGTGGGAATG | CAAACAACCCCAGATGCACA |
| 12 | LOC106436726(D) | CTCCAGCCTTTCTCGTGAGG | AGCTGTAATCAACGCTGTCATC |
| 13 | LOC106438719(U) | CCATCACTTACTTTCTCACCAGGA | TGGTCCGAGAGATGGATGACT |
| 14 | LOC106440999(D) | TAGCCGACAACTGGACAAGC | TCGGTCGCCATGTCAAATCA |
| 15 | LOC106447562(U) | GCATACGGATGATATGTTTGACCT | TCCCCGAGAAAGTGAACGTG |

^a^: “U” means up-regulated, “D” means down-regulated.

|  |
| --- |
