# Supplementary material for: Exploring the basis of 2-propenyl and 3-butenyl glucosinolate synthesis by QTL mapping and RNA-sequencing in Brassica juncea
Source: PLoS One. 2019 Oct 18;14(10):e0220597. doi: 10.1371/journal.pone.0220597 (PMC6799926; doi:10.1371/journal.pone.0220597)
Supplement: Attachment — Submitted filename: REVIEW.docx [file pone.0220597.s006.docx]

This manuscript constructed a genetic linkage map in *B. juncea*. Meanwhile, QTL mapping and RNA-seq were used to reveal the 2-propenyl and 3-butenyl GSLs synthesis. The results will be helpful for further fine mapping, gene cloning and genetic mechanisms of 2-propenyl and 3-butenyl GSLs in *B. Juncea*, which worth publishing in the PLOS one. But still some aspects need to be improved or considered in the further work：

1．Polymorphism comparison between the parental lines, makers, genetic map can be simply introduced in introduction and results; part;

2. The whole genome of *B. juncea* has been sequenced, how about the synteny relationships between the linkage map and sequencing results? It is better to show some comparative results because different *B. juncea* lines may have structural variations in certain regions on their genomes.

3. Transcriptome data analysis and processing can be briefly introduced, but with emphasis on describing transcriptome results and the differential expressed genes associated with those QTL results, especially those differential expressed genes involved into the 2-propenyl and 3-butenyl GSLs biosynthetic pathways;

4. The discussion part may be focused on the significance and application of the new QTL detected for breeding. How about the relationship between the QTL on LG08 and previous published ones?
